# 91 The use of process evaluations in physical activity interventions for older adults: a systematic review

**DOI:** 10.1093/eurpub/ckae114.116

**Published:** 2024-09-26

**Authors:** Michael Adams, Wendy Hardeman, Marie Murphy, Liz Simpson, Mark Tully

**Affiliations:** Ulster University, Belfast, Northern Ireland; University of East Anglia, Norwich, United Kingdom; Ulster University, Belfast, Northern Ireland; Ulster University, Belfast, Northern Ireland; Ulster University, Belfast, Northern Ireland

## Abstract

**Background and purpose:**

Development of effective physical activity interventions for older adults is important in eliciting positive health and well-being outcomes. Process evaluations of interventions are important in understanding implementation, mechanisms of impact and contextual factors that may influence these outcomes. However, it is unclear whether process evaluation frameworks are rigourously applied to physical activity interventions with older adults, particularly in relation to Medical Research Council (MRC) process evaluation guidance. This study aimed to systematically review the application and reporting of process evaluations of physical activity interventions for older adults against MRC process evaluation guidance.

**Methods:**

MEDLINE, EMBASE, CINAHL, AMED, PsycINFO, SPORTDiscus, OpenGrey, ProQuest Dissertations & Theses Database (PQDT) databases were searched. Process evaluation studies of physical activity interventions for older adults which measured at least two key MRC domains of implementation, mechanisms of impact and context were included. Two authors completed independent screening of title, abstract and full-text articles. A bespoke coding frame was developed to map and extract methods and findings on to key MRC process evaluation domains. Given the qualitative nature of the data, a narrative synthesis approach was undertaken.

**Results:**

17,002 studies were identified in the search, of which 27 were included. After mapping the methods of included studies against key domains of the coding framework; 21 assessed implementation, 27 assessed mechanisms of impact and 12 assessed context. 16 interventions reported the use of a process evaluation framework, 15 cited the use of a theoretical behavioural model and 7 reported the application of a logic model in intervention design. There were substantial differences in both the extent and quality of process evaluation methods. Such disparities were shaped in part by variations in the application of theoretical models and frameworks.

**Conclusions:**

When mapping the included studies against MRC process evaluation guidelines, this review presented inconsistencies in how process evaluations have been reported and findings suggest a lack of methodological rigour. Findings highlight the need to extend the scope and quality of process evaluations of physical activity interventions for older adults.

